# Efficacy of Transcutaneous Electrical Nerve Stimulation (TENS) for Management of Pain Associated With Hysteroscopy: A Systematic Review

**DOI:** 10.7759/cureus.70797

**Published:** 2024-10-03

**Authors:** Juliet Galtes, Rachel Siretskiy, Lauren Offield, Benny Esquenazi

**Affiliations:** 1 College of Medicine, Florida International University, Herbert Wertheim College of Medicine, Miami, USA; 2 Department of Obstetrics and Gynecology, Memorial Healthcare, Miami, USA

**Keywords:** acute pain management, obstetrics & gynecology, outpatient hysteroscopy, procedural pain control, transcutaneous electrical nerve stimulation, transcutaneous electrical nerve stimulation (tens)

## Abstract

Transcutaneous electrical nerve stimulation (TENS) therapy is a simple, non-pharmacologic, and non-invasive analgesic intervention that involves the application of electric currents over an area of pain to stimulate underlying nerves. The methodology behind TENS is based on theories of pain perception and processing such as the gate control theory, descending inhibition, and release of endogenous opioids. TENS has also been shown to play a role in the management of pain for gynecologic procedures such as hysteroscopy. Hysteroscopy is an increasingly popular diagnostic and interventional procedure, but its associated pelvic pain is a significant obstacle for patients. This systematic review aims to identify if TENS is an effective analgesic modality during hysteroscopy.

## Introduction and background

Transcutaneous electrical nerve stimulation (TENS) therapy is a pain control modality that utilizes electrical currents to target nerve stimulation within a specific area of the body. It has gained recent attention from the medical community due to its practicality, efficiency, and non-pharmacologic, non-invasive approach. Small electrode pads are placed on the skin overlying relevant dermatomes of the affected area, and the electrodes are wired to a compact, battery-powered machine. This TENS unit discharges electrical currents through the electrodes and stimulates underlying nerves to provide analgesia. The currents can be modified by users to set the amplitude, frequency, width, duration, and pattern to their liking [1].

In the realm of Obstetrics and Gynecology, TENS has been found to be an effective pain reliever in patients with dysmenorrhea and chronic pelvic pain syndrome [2,3]. TENS has also found success in laboring women and cesarean section operations [4,5]. Pain is the common obstacle shared between these pathologies and procedures, and hysteroscopies are no exception. Hysteroscopy is a minimally invasive procedure used for the visualization and treatment of intrauterine pathologies. There are two main types of hysteroscopy: diagnostic and operative. With diagnostic hysteroscopy, intracavitary uterine anomalies such as polyps, fibroids, and septa can be directly visualized and diagnosed. In operative hysteroscopy, these anomalies can be treated via polypectomy and curettage. Both consist of inserting a thin telescope with a light and camera at the end through the cervix and into the uterus. Additionally, both are associated with pelvic pain during or shortly after the procedure [6].

Pain during hysteroscopy can be attributed to various factors, such as direct manipulation of the cervix with a tenaculum, dilation of the cervix, insertion of the hysteroscope, stretching of the uterine cavity with the fluid medium, and irritation of the peritoneum caused by the accumulation of this fluid in the peritoneal cavity. To add on, cervical manipulation and uterine cavity distension promote the release of prostaglandins, which further augment pain [7]. The most common cause of hysteroscopy failure is pain [8]. Despite this, there are currently no commonly accepted guidelines for the management of pain associated with hysteroscopy.

## Review

Methods

This systematic review was performed in alignment with the Preferred Reporting Items for Systematic Reviews and Meta-Analyses (PRISMA) guidelines. The primary aim was to synthesize evidence related to the efficacy of transcutaneous nerve stimulation as a pain relief tool for hysteroscopy, providing a comprehensive overview of the existing literature. We conducted our search through PubMed, the Cochrane Central Register of Controlled Trials, and Embase. The search strategy was developed in consultation with a librarian experienced in systematic reviews. Keywords and Medical Subject Headings (MeSH) terms of (transcutaneous nerve stimulation OR transcutaneous nerve stimulator OR transcutaneous nerve stimulators OR transcutaneous electric stimulation OR electrostimulation OR transcutaneous electrical neurostimulation) AND (hysteroscopy OR hysteroscopic) were used to identify relevant studies as of August 2024. 

Duplicates were removed manually. Then, two independent reviewers screened the titles and abstracts of the studies against the eligibility criteria. Studies were eligible for inclusion if they were randomized control trials, their populations were women of any age undergoing hysteroscopy for any indication, transcutaneous nerve stimulation was the independent variable, and the dependent variable was pain modulation. We excluded studies that concurrently discussed TENS use for other pelvic pathologies and where TENS use was aggregated with other methodologies for pain reduction. Full-text articles were retrieved for potentially relevant studies, and disagreements were resolved through discussion or consulting a third reviewer.

Data were extracted independently by two reviewers using a standardized data extraction form. The extracted data included the year the study was conducted, authors, country of study conduct, age of patients in the study population, sample sizes of the intervention group/control group/placebo group, placement of the electrodes, frequency at which stimulation was given, timing of TENS use (before, during, after the procedure), differences in baseline characteristics of study populations, criteria by which outcomes were determined, pain assessment tool employed, and the main findings of the study. 

Results

**Figure 1:**
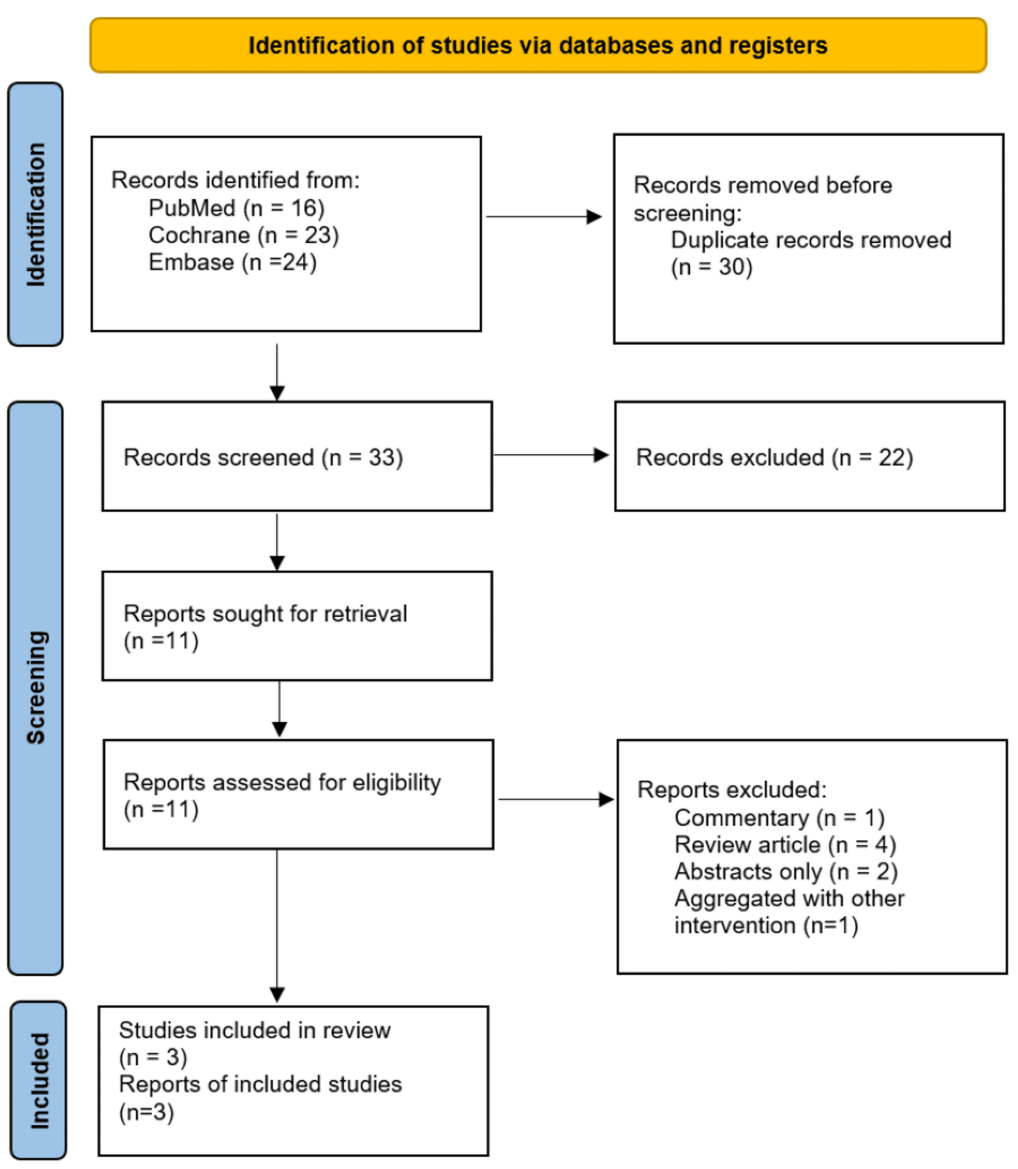
The Preferred Reporting Items for Systematic Reviews and Meta-Analyses 2020 flow diagram From Page et al. [9]

The initial search utilizing the MeSH strategy across PubMed, Cochrane, and Embase yielded 63 articles. Of these, 16 were identified on PubMed, 23 on Cochrane, and 24 on Embase. After removing 30 duplicates, 33 articles were screened based on titles and abstracts. Of these articles, 11 were identified as potentially relevant and underwent full-text review. Of these studies, one was excluded as it was a commentary on another article, four others were identified as being review articles, two were solely abstracts with no associated article, and one was excluded due to having an aggregate dependent variable. Ultimately, three studies met the inclusion criteria. The study selection process is detailed in the PRISMA flow diagram (Figure 1).

A total of three studies were included, and the characteristics of these studies are outlined in Table 1.

**Table 1:** Characteristics of the included studies cm, centimeter; Hz, hertz; PACU, post-anesthesia care unit; RCT, randomized controlled trial; TENS, transcutaneous electrical nerve stimulation; VAS, visual analog scale

Authors	Years of publication	Country	Study design	Placement of electrodes	Frequency of stimulation	Timing of TENS use (before, during, and after procedure)	Baseline characteristics	Pain scale classification	Outcome measurements	Outcomes
De Angelis et al. [10]	2003	Italy	RCT	Abdomen in the middle of the line joining the iliac spine to the pubic tubercle	100 Hz	Before and during procedure	No significant difference	No pain: VAS score of 0; mild pain: VAS score of 1-3 cm; moderate pain: VAS score of 4-7 cm; severe pain: VAS score of 8-10 cm	Primary outcome: pain reduction based on 10 point scale; secondary outcome: side effect presence, changes in hemodynamic parameters	1. TENS group experienced a significantly lower level of pain during hysteroscopy; 2. No differences in side effects were observed
Lisón et al. [11].	2016	United States	RCT	Parallel to the spinal cord at the T10-L1 and S2-S4 levels	80-100 Hz	Before and during procedure	More nulliparous women in the intervention group	Mild pain: VAS score of 0-3 cm; moderate pain: VAS score of 4-6 cm; intense pain: VAS score of 7-10 cm	Primary outcome: self-reported pain intensity (0-100 mm); secondary outcome: duration of the procedure, vital parameters, vasovagal symptoms, and participant satisfaction index (0-10 rating scale)	1. TENS group reported significantly lower pain scores compared to the control group; 2. No difference in the presence of vasovagal symptoms between the two groups
Platon et al. [12]	2020	Sweden	RCT	Positioned over the area where the patient experienced pain, which was most frequently over dermatome TH12	80 Hz	After procedure	No significant difference	Mild pain: VAS score of <5; moderate pain: VAS score of 5-6; severe pain: VAS score of 7-10	Primary outcome: pain reduction after arrival to PACU; secondary outcome: time spent in PACU	1. TENS group had a statistically significant reduction in pain scores; 2. Patients in the TENS group had shorter duration in the PACU

As evidenced in Table 1, research surrounding the use of TENS during hysteroscopies is scarce. Nonetheless, careful interpretation of the existing literature is paramount to crafting a thorough review and paving the way for future research. The first randomized controlled trial on this topic was conducted in 2003 by De Angelis at "La Sapienza" University in Rome, Italy [10]. The trial featured 142 women undergoing office hysteroscopy. Among the women, there were no significant differences in baseline characteristics (mean age, vaginal delivery percentage (%), cesarean section (%), menopause (%), years from menopause, hormone replacement therapy (%), cervical diathermy (%)) except for nulliparous (%), where the intervention group had significantly more nulliparous women than the control group (<0.05). The intervention group, composed of 71 women, received TENS therapy five to 10 minutes before starting the hysteroscopy procedure by means of two electrodes placed on the patient's abdomen in the middle of the line joining the iliac spine to the pubic tubercle. The TENS device functioned at a frequency of 100 Hertz (Hz), and the patients had the freedom to decrease or increase amplitude as needed. The control group was also composed of 71 women, and they did not receive any form of pain control during hysteroscopy. To measure the degree of pain experienced during the procedure, patients were asked to rate their pain on a visual analog scale (VAS) using four criteria: 0 = no pain, 1-3 cm = mild, 4-7 cm = moderate, and 8-10 cm = severe pain. Within the intervention group, intraoperative pain levels were measured at basal level TENS stimulation and after they had increased the amplitude of the device. At the end of the procedure, patients were also asked about the occurrence of side effects, including shoulder pain, nausea, vomiting, and dizziness. Results of the study showed significantly lower mean pain scores in the intervention group compared with the control group (group A mean, 3.71 ± 2.06; group B mean, 5.07 ± 2.03; p < 0.0004). To add on, significantly lower pain scores were reported after patients had increased device amplitude compared to basal level amplitude (basal TENS mean, 6.16 ± 1.8; activated TENS mean, 3.71 ± 2.06; p = 0.0001). There were no statistically significant differences between groups with regard to side effects. In essence, De Angelis proved that TENS was an efficient and safe method of relieving pain during office hysteroscopy. 

In 2016, a randomized, double-blind, placebo-controlled study conducted in Spain by Lisón showed similarities to the De Angelis study in regards to both methods and results [10,11]. Although a smaller study, consisting of only 138 women, Lisón sought to evaluate the pain-relieving effect of TENS during office hysteroscopy. There were no significant differences in baseline participant characteristics. Patients were divided into three groups, with 46 patients in each group: active TENS, placebo TENS, and control. Similar to the De Angelis study, the TENS device featured a frequency of 80-100 Hz, and amplitude was self-adjusted by patients [10,11]. In the active TENS group, patients received TENS therapy five minutes before the start of the hysteroscopy and through the duration of the procedure via two sets of two self-adhesive electrodes placed parallel to the spinal cord at the T10-L1 and S2-S4 levels. Participants in the placebo TENS group were connected to the TENS device the same way as participants in the active TENS group but received no electrical stimulation. Much like the De Angelis study, participants in the control group underwent hysteroscopy with no forms of sedation or analgesia [10,11]. Patients used a 100-mm-long horizontal line VAS where 0 mm = no pain and 100 mm = worst possible pain at several stages of the procedure (baseline, hysteroscope entry, endometrium contact, biopsy, and five minutes after end of procedure) to self-report their pain intensity. Results showed that compared to the placebo TENS and control group, participants in the active TENS group reported a significant decrease in pain scores during all stages of the procedure (entry: -11 mm, 95% confidence interval (CI) -17 to -5; contact: -21.9 mm, 95% CI: -30 to -13.9; biopsy: -30.5 mm, 95% CI: -47.1 to -13.8, p < 0.001). 

The latest study, conducted in 2020 by Platon, was an even smaller study consisting of 74 women [12]. Although the study aimed to assess the analgesic effects of TENS on patients undergoing hysteroscopy, their design was very different from those of De Angelis and Lisón [10,11]. In this study, Platon assessed TENS use in the postoperative anesthesia unit (PACU) in women experiencing pain (reported as ≥3 on a VAS) after undergoing hysteroscopy in the operating room under general anesthesia. Participants were divided into two groups: the TENS group and the opioid group. In the TENS group, electrodes were positioned over the area where the patient experienced pain, which was most frequently over dermatome TH12. Patients did not have the opportunity to adjust amplitude to their liking and instead received an amplitude of 40-60 milliamperes (mA) or 19-38 mA if the prior was too strong. TENS therapy was administered for 1 minute, after which pain levels were measured via a VAS where a score of seven to 10 was defined as severe pain, five to six as moderate pain, and less than five as mild pain. If adequate pain relief (VAS < 3) was not obtained after one minute of TENS therapy, patients were given up to two more episodes of TENS therapy, after which they were given opioid medications. On the other hand, the opioid group received 5.3 milligrams (mg) of IV morphine. Pain levels were assessed using a VAS after 10 minutes of opioid administration. If adequate analgesia (VAS < 3) was not achieved, primary analgesics (i.e., morphine, paracetamol, NSAID, or tramadol) were administered according to local clinical guidelines. If analgesia was not achieved 30 minutes after the start of treatment, they received TENS therapy. Results showed that upon arrival to PACU, there were no significant differences between the groups in terms of pain intensity. Both groups reported significant pain relief, with a decrease in VAS score from 5.6 at arrival to 1.4 at departure from the PACU in the TENS group (p < 0.001) and a decrease in VAS score from 5.1 at arrival and 1.3 at departure from the PACU in the opioid group (p < 0.001). In essence, the study proved that both treatments with high-intensity, high-frequency stimulation using TENS and pharmacological treatment with IV opioids are effective for postoperative pain relief after gynecologic hysteroscopy. 

There are many similarities, differences, and limitations surrounding the studies listed in Table 1. Both De Angelis and Lisón assessed the use of TENS in office hysteroscopy compared to a control group [10,11]. Lisón also included a placebo group, which may have been helpful in assessing the psychological influence of TENS therapy [11]. On the other hand, Platons study presents many differences and confounding variables that make it difficult to properly compare its results to prior studies [12]. For example, in the Platon study, participants underwent hysteroscopy in the operating room under general anesthesia. This is not an in-office procedure, and the effects of anesthesia and perioperative medications may affect perceptions of pain. In addition, the study applied TENS in the PACU, not during the procedure. Perhaps one of the reasons why the Platon study did not find significant results between groups is because they were comparing TENS therapy to morphine administration and not a control group. In this sense, it is difficult to assess whether TENS therapy provided an analgesic effect at all. 

Other factors that require review are the type of hysteroscopy instruments used and intrauterine procedures conducted during hysteroscopy. In addition, since hysteroscopy is a user-dependent procedure, it is difficult to assess how user experience and technique may have affected study outcomes. In the De Angelis study conducted in 2003, all hysteroscopies were performed using a 4-mm telescope with a 30° fore-oblique lens and a 5 mm thick outer diagnostic sheath [10]. This differs from the one used in the Lisón study, which was a Richard Wolf Panoview Plus 12° 3.5-mm hysteroscope (Richard Wolf GmbH, Knittlingen, Germany) with a 5-mm-thick outer diagnostic sheath [11]. Since entry into the uterine cavity through the cervical canal is a source of pain during hysteroscopy, it is important to assess whether these differences in hysteroscope diameter had an influence on results. Regardless, both studies found supporting evidence for the analgesic effects of TENS therapy compared to standard control groups. Finally, procedures during the hysteroscopy, such as polyp removal or biopsies, are also important factors that may influence the perception of pain. De Angelis did not report any procedures during hysteroscopy, but Lisón and Platon did [10-12]. Lisón specifically mentioned the conduction of biopsies, which are inherently more painful than a simple diagnostic hysteroscopy [11]. Despite performing more painful procedures, Lisón still found TENS therapy to be an effective analgesic method when compared to a control group.

Discussion

TENS Proposed Mechanism of Action

TENS relies on the general understanding and physiology of pain perception. Peripheral noxious stimuli are sensed by nociceptors and conveyed through primary neurons, A-delta or C fibers, that synapse with secondary neurons at the substantia gelatinosa (SG) in the dorsal spinal cord. These fibers release glutamate and substance P, respectively, to generate an action potential in secondary neurons, which ascend contralaterally through the spinothalamic tract of the spinal cord and synapse with tertiary neurons in the thalamus. Tertiary neurons then project to the postcentral gyrus of the parietal lobe and result in conscious awareness of pain [13]. A separate pathway called the dorsal column medial lemniscus (DCML) is used for the perception of touch, pressure, vibration, temperature, and other non-noxious stimuli. These travel through primary neurons, A-alpha and A-beta fibers, up the ipsilateral dorsal column of the spinal cord and synapse with second order neurons at the medial lemniscus of the medulla oblongata. Second order neurons decussate before continuing up and synapsing with third-order neurons in the ventral posterolateral nucleus of the thalamus. Tertiary neurons then project to the postcentral gyrus of the parietal lobe to create conscious awareness of touch [14].

The analgesic effects of TENS therapy are based on theories of pain perception and processing, such as the gate control theory, descending inhibition, and release of endogenous opioids. The gate control theory, proposed in 1965 by Patrick David Wall and Ronald Melzack, suggests that the spinal cord has a neurological mechanism by which it blocks or allows the passage of pain signals to the brain [15]. This mechanism relies on the DCML pathway, specifically on collaterals of A-alpha and A-beta fibers that synapse with inhibitory interneurons in the SG. These interneurons release enkephalins, an inhibitory neurotransmitter that binds to opioid receptors on A-delta and C fibers, preventing their release of excitatory neurotransmitters and decreasing the relay of pain signals. By this process, rubbing, using an ice pack, or a TENS unit on a painful area can decrease pain perception. In TENS therapy, electrical currents sequentially stimulate mechanoreceptors on the skin, neurons of the DCML pathway, and inhibitory interneurons in the SG, which ultimately inhibit pain signals [16]. Other theories postulate that TENS utilizes a descending inhibitory pathway and can even increase the release of endogenous opioids [17].

Optimal Electrode Placement

With regard to electrode placement, there is no consensus, and studies differ in their methods. In the study conducted by De Angelis [10], electrodes were placed between the iliac spine and pubic tubercle on each side. This area belongs to dermatome TH12, the placement used in the Lisón study [11]. This, however, differs from the electrode placement used in the Platon study [12], where two sets of 7 × 13 cm rectangular electrodes were placed parallel to the spinal cord at the T10-L1 and S2-S4 levels. The rationale for their differing placement is based on prior reports that greater pain relief occurs when electrodes are placed within the dorsal horn's receptive fields [1]. Further research is needed to determine the most effective electrode placement for analgesic effects during hysteroscopy. 

Optimal Frequency of Stimulation

TENS classification is made on the basis of the output, measured in frequency, of the device rather than the physiological response elicited by this output. This is divided into three subsects: conventional (low intensity, high frequency), acupuncture-like (high intensity, low frequency), and intense TENS (high intensity, high frequency). Each technique has different intentions for managing pain control. The conventional method stimulates a large diameter at a low-threshold noxious stimulus with the intent of inhibiting the activity of second order nociceptive transmission in a selected dermatome. The acupuncture-like method is utilized by those who do not respond to the conventional. It aims to stimulate a small diameter of higher threshold peripheral afferents with the intent of activating the descending inhibitory pathway. Lastly, the intense method is typically reserved for pain management in short bursts as it targets a small diameter of high threshold cutaneous afferents to block nociceptive transmission to peripheral nerves [18].

Additionally, while low-frequency (<10 Hz), high-intensity TENS is also utilized in clinical practice, it has mostly been shown to have efficacy in individuals with chronic pain, whereas high-frequency (80-100 Hz), high-intensity TENS has better applicability for acute pain since it activates gate control by stimulating A-beta fibers [19].

In the case of the studies described, they all utilized the intense method (high-frequency, high-intensity), demonstrating that this range is most effective for targeting the acute pain experienced during the hysteroscopy procedure [10-12]. However, one of the limitations of these studies is the difficulty in establishing the most effective frequency for optimal pain management. On the one hand, the TENS device utilized in Lisón's study delivered frequencies between 80-100 Hz randomly, while Platon utilized a consistent 80 Hz frequency, and De Angelis utilized a consistent 100 Hz frequency [10-12]. While both classify as the intense method, this leaves the question of the frequency at which pain management for hysteroscopy is optimized. Since none of the studies examined subjective pain reports on the basis of differing frequencies within the intense range, there is still a need for further exploration of the optimal frequency. 

Optimal Timing of Placement

Much like the uncertainty that surrounds proper electrode placement, significant confusion exists regarding the optimal timing for electrode application. The efficacy of TENS can be influenced by when electrodes are applied, with varying results depending on whether they are placed before, during, or after a procedure. The timing of electrode placement can affect the intensity and duration of pain relief, adding an additional layer of complexity to pain management strategies. For example, De Angelis placed their electrodes five to 10 minutes before the start of hysteroscopy and removed them after the patient was well enough to sit up [10]. Similarly, Lisón applied the electrodes five minutes before the start of the procedure and throughout the duration [11]. This differs from Platon, where patients received TENS therapy post-operatively in the post-anesthesia care unit for only one minute and received another minute if pain relief was insufficient [12]. This variability underscores the need for further research to establish clear guidelines on both the placement and timing of electrodes to maximize therapeutic outcomes and enhance patient comfort.

Efficacy for Pain Reduction in Hysteroscopy

Although studies have demonstrated that TENS has proven pain-relief in various gynecologic pathologies and procedures, its degree of efficacy remains in question. One of the barriers to accurately assessing its efficacy is measuring pain reduction. Most studies, including De Angelis, Lisón, and Platon, use VASs to measure pain [10-12]. However, the scores that correspond to mild pain, moderate pain, and severe pain differ. For example, De Angelis utilized a 10 cm VAS where severe was defined as 8-10 cm, moderate as 4-7 cm, mild as 1-3 cm, and 0 cm as no pain [10]. Lisón used the same scale, but severe pain was defined as 7-10 cm, moderate as 5-6 cm, and mild as less than 5 cm [11]. These inconsistencies can lead to different interpretations of pain and the exact efficacy of TENS. In addition, there exist additional physiologic factors that affect the perception of pain and are difficult to take into account. These include anxiety, culture, social support, and emotional state. Positive emotions usually decrease perceived pain, while negative emotions intensify perceived pain [20]. The perception of pain remains a complex and evolving field of study that requires further research. Despite advancements in neuroscience and pain management, the exact mechanisms through which pain is perceived and processed are not entirely understood.

## Conclusions

This systematic review examined the efficacy of TENS as a potential non-invasive pain management tool that can be utilized for the management of pain associated with hysteroscopy. The findings indicate that TENS is effective in significantly reducing subjective pain perceptions in patients undergoing hysteroscopy. However, variability in frequency used, placement of the probes, and overall small sample sizes highlight the need for further large-scale, randomized controlled trials to establish standardized guidelines and confirm the long-term benefits of TENS in the context of pain management for hysteroscopy.
